# Promoting Human Nutrition and Health through Plant Metabolomics: Current Status and Challenges

**DOI:** 10.3390/biology10010020

**Published:** 2020-12-31

**Authors:** Wenli Sun, Zican Chen, Jun Hong, Jianxin Shi

**Affiliations:** Joint International Research Laboratory of Metabolic & Developmental Sciences, School of Life Sciences and Biotechnology, Shanghai Jiao Tong University, Shanghai 200240, China; sunwenli@sjtu.edu.cn (W.S.); chenzican@sjtu.edu.cn (Z.C.); hongjun0699@sjtu.edu.cn (J.H.)

**Keywords:** bioactive compounds, food safety, human health, human nutrition, metabolomics

## Abstract

**Simple Summary:**

This review summarizes the status, applications, and challenges of plant metabolomics in the context of crop breeding, food quality and safety, and human nutrition and health. It also highlights the importance of plant metabolomics in elucidating biochemical and genetic bases of traits associated with nutritive and healthy beneficial foods and other plant products to secure food supply, to ensure food quality, to protect humans from malnutrition and other diseases. Meanwhile, this review calls for comprehensive collaborations to accelerate relevant researches and applications in the context of human nutrition and health.

**Abstract:**

Plant metabolomics plays important roles in both basic and applied studies regarding all aspects of plant development and stress responses. With the improvement of living standards, people need high quality and safe food supplies. Thus, understanding the pathways involved in the biosynthesis of nutritionally and healthily associated metabolites in plants and the responses to plant-derived biohazards in humans is of equal importance to meet people’s needs. For each, metabolomics has a vital role to play, which is discussed in detail in this review. In addition, the core elements of plant metabolomics are highlighted, researches on metabolomics-based crop improvement for nutrition and safety are summarized, metabolomics studies on plant natural products including traditional Chinese medicine (TCM) for health promotion are briefly presented. Challenges are discussed and future perspectives of metabolomics as one of the most important tools to promote human nutrition and health are proposed.

## 1. Introduction

Plants are well known as the factories producing plenty of natural compounds, the number of which is estimated to be more than 200,000 [1]. Producing such a huge diversity of metabolites with different structures by plants is one of their unique adaptive features; unlike animals, plants are sessile organisms [2]. These metabolites, known as primary and/or secondary metabolites, are very important in plant growth, development, and response to the surrounding environment [3,4]. They are also important resources of foods, medicines, and industrial raw materials for humans [5,6,7]. Understanding the diversities, functions, and pathways of these plant-derived natural metabolites, is, thus, fundamental not only for food security and food nutrition but also for the production of pharmaceuticals and novel materials through plant metabolic engineering. On the other hand, understanding the responses of humans to various dietary or herbal derived molecules including biohazards and active herbal components is vital for human health.

Metabolomics is an important component of post-genomic omics science that deals with the comprehensive chemical analysis of the entire metabolites, so-called the metabolome, in a particular biological system, for example, a cell, a tissue, or an organism at a specific developmental stage or under an induced circumstance. Since plants produce more chemicals than any other organisms such as microorganisms and animals [1], metabolomics is of special importance in the elucidation of plant chemical arsenals and their functional properties. Various instrumentations have been used for metabolomics studies, and the advances in instrument and computation development have been reviewed elsewhere [1,8,9,10,11,12,13,14]. In brief, currently, the analytical technology of metabolomics is based on either mass spectrometry (MS) or nuclear magnetic resonance spectrometry (NMR), supported by bioinformatics [15,16]. It is worthy to note that no single metabolomics method can determine all metabolites in a sample; indeed, such a method may never exist. Therefore, metabolomes revealed by any combined metabolomics methods reflect only the tip of the iceberg of a cell, tissue, or organism. Nevertheless, the power (accuracy and coverage of metabolite measurements) of current metabolomics and bioinformatics is strong enough to support its application in both basic and applied research, which significantly facilitates crop breeding for yield and quality [15,17,18,19,20], plant-derived drug discovery and production [21,22,23], safety assessment of genetically modified (GM) crops [24,25,26], and biohazards derived from plants [27,28].

Different from genomics, transcriptomic, and proteomics, metabolomics represents the final measurable responses (biochemical features/phenotypes) of the molecular function of a cell, which cannot be directly deduced from any other omics including genomics, transcriptomic, and proteomics, due mainly to the complicated regulatory mechanisms occurred at both transcriptional and translational levels [27]. Therefore, among different omics methods, metabolomics is the most predictive one, which provides the closest link between genotype and phenotype. Metabolomics can be particularly useful in crop improvement for yield, quality and safety; the underlying logic is that almost all editable and herbal crop parts, such as seeds, tubes, and roots, are primarily sinks of various metabolites from the upstream gene function and protein activity. In this case, plant metabolomics can be useful to identify genes that are associated with crop quality and safety under both normal and stress conditions to increase crop production and to meet the needs of food supply for increasing populations particularly under climate change conditions [15,17,28,29,30]. In addition, plant metabolomics helps to better understand biochemical bases of what we eat from dietary foods, how metabolites change along the process of production, storage and transportation, and processing and cooking, and thus to design healthier foods, to overcome obesity and malnutrition problems that affect human health worldwide [7,20,29,31,32,33,34]. Furthermore, plant metabolomics aids in the discovery of new natural product-based drugs, the quality evaluation of herbal medicines, and pharmaceutical production, thus benefiting human health [21,23].

This review discusses approaches, core elements, and their applications of metabolomics in crop improvement on various aspects relevant to human nutrition and health. Challenges are identified and a future perspective is proposed for the future application of plant metabolomics as an important tool to improve the quality of human life.

## 2. Approaches of Metabolomics

Current metabolomics approaches, depending largely on experimental objectives, namely, biological questions, are generally classified into targeted and non-targeted metabolomics, differing significantly in metabolic coverage and quality [8,35,36]. Targeted metabolomics focuses on the investigation of a limited number of metabolites or a single class of compounds of known biological relevance, using dedicated and optimized methods, which are primarily used for comparative metabolite profiling of known metabolites. Non-targeted metabolomics investigates hundreds or thousands of multiple classes of known and unknown compounds (with no identical quality for all covered metabolites) or identifying new metabolites/pathways. In between, the metabolite-fingerprinting approach provides the widest profile coverage; however, neither identities nor levels of metabolites are necessarily resolved. In this review, we focus on non-targeted metabolomics/metabolite profiling, the most used, so-called discovery-based metabolomics approach [36].

## 3. Standardization of Plant Metabolomics Studies

**Figure 1:**
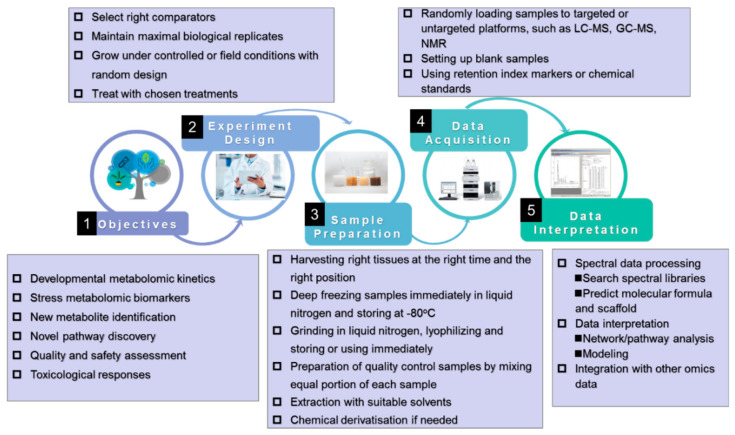
An overview of the pipeline of a plant metabolomics study. LC-MS, liquid chromatography mass spectrometry; GC-MS, gas chromatography mass spectrometry; NMR, nuclear magnetic resonance.

Although the pipeline of plant metabolomics (Figure 1), which includes three interrelated steps, namely sample preparation, data acquisition, and data mining, can be readily found elsewhere in the literature, the importance of the standardization of plant metabolomics studies is not well recognized or popularized despite the presence of several recommendations [37,38,39].

Regardless of the analytical tools used, all steps of the metabolomics should be standardized to ensure direct comparison of data generated from different laboratories at different times. Standardization of metabolomics steps is also an absolute requirement for building metabolomics databases. Thus, there is a need to introduce and implement quality assurance (QA) and quality control (QC) to ensure high quality of data acquired, reported in scientific publications, and housed in databases for non-targeted metabolomics as do in targeted metabolomics [36,37,39]. So far, recommendations for experimental design, quality assurance and control, and, standard operating procedures (SOPs) to ensure each step is executed meticulously and always in the same manner, have been proposed for non-targeted plant metabolomics [37,39,40,41]. These recommendations and documents help to avoid errors in operation along with the analysis and misinterpretation of results and facilitate comparisons among datasets in plant metabolomics studies, which lays a solid foundation for its application in crop improvement and human health promotion.

It must be highlighted that, unlike genomics, various internal and external factors substantially impact the outputs of metabolomics analysis. Sampling, the first step of plant metabolomics, fundamentally affects the quality of metabolomics data, thus, special attention has to be paid to harvesting and extraction. Harvesting should be conducted quickly, at the right time, from the right tissue, and at the right position; plant metabolites fluctuate with a day-night cycle, tissues, developmental stages, and positions in the plant; they are easily altered by surrounding environments during harvesting [38]. Once harvested, samples have to be frozen immediately in liquid nitrogen. Any slight delay in freezing the sample or any improper sample freezing can bring about significant changes, misleading data interpretation; thus, bringing a liquid nitrogen tank to the sampling site is the best choice. Freshly frozen samples can be used directly for metabolomics analysis or stored at −80 °C for later use. In most cases, frozen samples are lyophilized before metabolite extraction. For sample extraction, extraction methods used in phytochemical studies are suitable for plant metabolomics, however, the choice of extraction methods, including the solvent, the ratio of solvent and sample, and the duration of extraction, depends greatly on the objectives of the study and the metabolomics approaches used. Unlike LC or NMR systems, an additional step, chemical derivatization, is necessary for GC to increase the volatility of samples, which is required for analyzing many metabolites such as amino acids, fatty acids, sugars, and organic acids.

Special attention should also be paid to data normalization to minimize batch-to-batch variation. It is especially important for large-scale metabolomics studies, where integration data from independent batches of samples would be very difficult if batch variations were present [42]. To minimize batch variation derived from sample extraction, an internal standard is often added to the sample at a critical analysis step [42,43]; sometimes multiple internal standards are also used [44]. QC samples prepared by mixing equal volumes of all investigated biological samples [42,45] and synthetic mixtures of authenticated reference compounds play important roles in the determination of the variance of a metabolite feature, facilitating the removal of batch variations and other technical variations such as machine drifts and noise [42].

## 4. The Applications of Plant Metabolomics in Crop Improvement for Human Nutrition and Human Health

Understanding genetic and biochemical mechanisms underlying trait formation in crops under both normal and stressed conditions form the basis of the application of metabolomics in crop improvement for human nutrition and health [3,5]. Metabolomics, combined with other omics, facilitates crop improvement by linking a specific metabolite or a metabolic pathway more rationally with a trait of nutritive and healthy importance, through interspecies metabolomics comparison [31,43,46,47], metabolite quantitative trait loci (mQTLs) [48,49], and metabolome-based genome-wide association study (mGWAS) [50,51,52,53]. mGWAS alone or combined with phenotype GWAS (pGWAS) significantly increases the power of metabolomics in the successful identification of candidate causing genes of important traits in crops, such as rice [48,54], maize [55,56], wheat [49,52,57], tomato [58,59], and other crops [18]. The below sections will review relevant achievements in the past two decades.

### 4.1. Plant Metabolomics and Natural Variations of Nutritional and Quality Relevant Metabolites and Underlying Genetic Mechanisms

Crop derived foods are important sources of human nutrition and health. We are always curious about what kinds of nutritional metabolites are present in crop derived foods, to what degree the natural variations of nutritional metabolites occur, and how to manipulate the metabolic flow for metabolic engineering; plant metabolomics provides us with a useful platform to look for answers, which greatly expands and updates our relevant knowledge. Understanding of natural variations and underlying mechanisms as well as factors governing temporal and spatial change patterns is fundamental for the identification of key metabolites for rational food quality design on nutritional and sensory quality of edible crop parts, including seeds, fruits, and tubers. Plant metabolomics has been widely used to investigate metabolomes of mature and developing seeds in several major food crops, particularly in rice [60,61,62,63,64], allowing us to understand what we eat, how they are formed, and how they are changed in the process of food crop development and food processing. Plant metabolomics studies have been also employed to identify common and/or specific macro- and micro-nutrients and quality associated components in food crops [32,64,65,66,67,68,69,70,71], their availability after cooking [70,72,73], and to discover biomarkers for future crop quality improvement. Comparative metabolomics strategies have revealed the convergent or divergent evolution of metabolism within or across plant species [47,56,63,74,75] and its mechanisms [76], which lays a solid base for crop improvement in the contest of nutrition and quality. The current review focuses mainly on major crop species.

#### 4.1.1. Rice

Large-scale metabolic profiling (GC-MS and UHPLC-MS/MS) in seeds of 100 *japonica* and *indica* cultivars identifies 121 rice grain metabolites, and reveals significantly diversified metabolomes between subspecies, reflecting not only a different local adaptive response but also a divergent nutritional value of their grains. For example, as compared with *indica*, *japonica* seeds contain lower levels of γ-tocopherol, γ-tocotrienol, and pyridoxate, but higher levels of phytic acid, gluconate, and nicotianamine [60]. However, mechanisms underlying metabolomic variations between these two rice subspecies remain unrevealed due to the lack of corresponding genomic data. Further metabolomics analysis (GC-MS and UHPLC-MS) on developing rice grains identifies 214 rice grain metabolites and demonstrates cultivar-dependent metabolic kinetics, pointing out that the grain-filling stage is the key time point that can be modulated to determine variable metabolites including nutritional compounds in rice grain [62]. Comparing metabolic kinetics between flag leaves (207 metabolites identified) and grains (121 metabolites identified) in rice finds that cultivar dependent metabolomic variations in leaves significantly impact metabolomics variations in grains [77], which is supported by another metabolomics analysis using an NMR platform [66]. Metabolomics (GC-MS and UHPLC-MS/MS) has been used to elucidate nutritional traits of newly released rice cultivars as well, in which the giant embryo rice “Shangshida No. 5” is found to contain more nutrients and bioactive compounds (such as reduced arabinose, β-alanine, glutathione, VB6, xylitol, and xylose) in addition to previous known GABA than its control cultivar “Chao2-10” [64], rendering “Shangshida No. 5” a suitable cultivar for potential rice improvement for nutritional quality. In addition to investigating the metabolome and the metabolic natural variation, many studies also go deeper to explore its underlying genetic mechanisms. Metabolic profiling (LC-MS-MS) in 38 rice varieties identifies 91 flavonoids, more in rice leaves and less in grains, and also shows that grain flavonoids are profoundly variable between *indica* and *japonica*, which is likely controlled by the sequence polymorphisms in flavonoid biosynthetic genes [53]. Metabolomics analysis (UPLC-MS-MS) combined with transcriptomics in 27 rice breeding lines indicates that resistant starch, non-starch polysaccharides in the cell wall, and flavonoids are important resources to be targeted to develop rice grains with slowly digestible starch to prevent and treat non-communicable disease such as diabetes mellitus and obesity in people on rice-based dietary [78,79]. Comparative metabolomics analysis (UPLC-MS-MS) on rice bran across 17 diverse cultivars reveals 71 significant variable rice bran metabolites, among them are some nutritious compounds of human health importance (including γ-tocopherol, β-tocopherol, and γ-tocotrienol) and over 500 potential causative genes, establishing a rice gene–metabolite relationship for rice bran traits breeding [29]. Untargeted lipidomics uncovers clear variations in lipid profiles between waxy and non-waxy grains and the association of putative lipids with amylose-lipid complex and amylose contents, important traits relevant to cooking and eating quality and nutritional quality [80]. Using combined exhaustive “multi-omics”, including metabolomics (GC-MS), transcriptomics, and label-free shotgun proteomics, variable compartmentalization of nutritionally relevant pathways is found between the endosperm and the embryo in *Nipponbare*, which provides further directions for attempts in rice nutritional quality improvement at the organ level [71].

#### 4.1.2. Maize

Metabolomic analysis (GC-MS and UHPLC-MS/MS) in seeds of 13 inbred lines in maize identifies 210 kernel metabolites and shows that many metabolites are negatively correlated to the antinutritional metabolite phytic acid, which provides a potential strategy to breed maize with lower phytic acid. It also finds that levels of antioxidant dihydrokaempferol, bioactive compound costunolide, precursors of vitamins, and nutritious substances are highly variable among different lines, implying the potential for breeding towards improved kernel quality and nutrition [81]. Comparing transcriptomic and metabolomics (GC-MS and UHPLC-MS-MS) dynamics in kernels between the colored and white maize discovers a remarkable up-regulation of anthocyanin and phlobaphene pathway in colored maize, and links primary metabolism with kernel coloration, providing a novel direction to improve nutritional compositions particularly secondary metabolites in maize [63]. Comparative metabolomics (GC-MS) further highlights the existence of active metabolites and pathways associated with oil content in the embryos, which paves the way for manipulating seed oil compositions in maize [67]. Metabolic profiling (LC-MS-MS) of 702 maize genotypes grown at different locations identifies 983 metabolite features, and mGWAS pinpoints out 1459 significant loci, and re-sequencing combined genetic studies confirms the function of five of these loci, which will accelerate the rate of genetic improvement of kernel nutrition quality in maize [55]. Dietary vitamin A deficiency is the main cause of eye disease in sub-Saharan African children living on maize-derived foods. With the help of a metabolomics study (HPLC-MS), significant natural variation for carotenoid composition, including precursors of vitamin A in maize kernels is revealed, which could be used to ultimately solve vitamin A deficiency by producing maize grain with higher provitamin A levels using uncovered natural resources [82]. Using mGWAS, expression QTL (eQTL), linkage mapping, and co-expression analysis, 26 important loci are found to be associated with natural variation of oil concentrations in kernels of 369 maize inbred lines, which lays a solid base for marker-based breeding to improve oil quality and oil nutritional value in maize [83].

#### 4.1.3. Soybean

Soybean seeds contain important nutrient metabolites that play roles in human disease prevention; structures and quantities of them, however, they are significantly affected by genetic and environmental factors. Compared with rice and maize, metabolic profiling (GC-MS and UHPLC-MS/MS) in 29 soybean cultivars indicates more and higher contents of antioxidants flavonoids, implying an added nutritional value of soybean seeds to human foods. The identified top nine mostly variable metabolites include one precursor for ascorbate, four isoflavones, and phytic acid, indicating that understanding more of the metabolic variations in soybean will have a profound impact on nutrition and quality improvement via hybridization or metabolic engineering [84]. By metabolomics comparison (GC-MS) among lines with varying protein and oil concentrations and their parents, free asparagine, free 3-cyanoalanine, and L-malic acid, are found to be associated with variable seed protein and/or oil content in soybean, providing useful biomarkers for improvement of soybean seed composition and nutrition [85]. Proteins are important nutrient components in soybean seeds, the amount and the number of storage, allergen, and anti-nutritional proteins also show major and minor natural variations among wild and cultivated soybean plants [86], which cannot be detected directly by metabolomics. However, some of these can be solved by amino acid profiling combined with other omics [87].

#### 4.1.4. Wheat

Non-target metabolomics (GC-MS and UPLC-MS) identifies 432 primary metabolites and 2756 secondary metabolites in wheat grains, among them, 46 primary metabolites and 12 secondary metabolites can serve as biomarkers distinguishing wheat grains grown under the organic cropping system from those grown under non-organic cropping system [69]. Combined with the health risk assessment of heavy metals in grains grown under two cropping systems, populations consuming grains from organic cropping systems have higher non-carcinogenic and carcinogenic risks, strengthening the need to evaluate both nutrition and potential detrimental health effects before beginning organic farming [69]. Non-targeted metabolomics (GC-MS) identifies in total 55 compounds in grain metabolome of aneuploidy wheat lines and reveals genes associated with variations for branched-chain amino acids and trehalose [88], providing molecular markers for wheat breeding. A metabolomic investigation (GC-MS) finds that breeding selection impacts tremendously on 51 primary metabolites and that certain primary metabolites can shape the domestication of wheat subspecies. For example, changes in unsaturated fatty acids and amino acids are linked with the domestication of emmer and durum wheat, respectively, providing evidence for exploring wild and exotic germplasm for wheat quality and nutritional trait improvement [57]. Using combined metabolomics (LC-MS/MS) and mQTL analysis, the genetic architecture of the wheat kernel metabolome has been established, in which 1005 mQTLs are discovered and among them, 24 genes are responsible for the observed metabolites variations. There are clear genetic relationships between metabolites and agronomic traits [49], as found in rice [60], and two genes of them are found to be involved in the biosynthesis and modification of flavonoids that are associated with human health [49]. To understand better complex metabolite-trait association in wheat, mGWAS using 805 identified metabolites (LC-MS-MS) and 14,646 SNP markers among 182 wheat accessions identifies 1098 mGWAS associations, among them, 26 candidate genes are potential causative genes for 42 loci, and further validation studies on two candidate genes uncover the flavonoid decoration pathway in wheat kernel [52]. With plenty of high-confidence genes linked with metabolite variations and metabolites associated with agronomic traits, these studies lay solid foundations for metabolomics-based wheat breeding.

#### 4.1.5. Other Crops

Compared with the abovementioned major staple crops, much fewer metabolomics studies have been used to investigate the natural variation in metabolomes of minor grain crops, such as barley, sorghum, oat, and millet, so-called coarse grains, that contain comparable and sometimes superior nutritional values to rice and wheat. These grains are rich not only in essential amino acids, dietary fibers, minerals but also in bioactive compounds. The application of metabolomics is helping the world to unravel the molecular basis of nutritional traits for improvement in their acceptability and nutritional attributes. In barley, metabolomics identifies metabolic markers for barley malting [89], reveals natural metabolomic variations among 72 barley breeding lines and 20 quality traits [90], and demonstrates the potential for optimal production of essential amino acids and dietary fibers [91]. In sorghum, there are currently no particular metabolomics reports on grains, but, metabolic profiling (GC-MS) in leaf tissues of 11 inbreeding lines shows clear associations of both primary and secondary metabolites (such as chromogenic and shikimic acid) with photosynthesis and biomass [92]. In oat, metabolomics studies (GC-MS) using grains from 12 wild and 10 cultivated oat accessions recommend some wild species as potential sources of nutritional traits for breeding purposes and identifies metabolites that vary during domestication or differentiates wild oats from cultivars [93]. In addition, comparative metabolomic profiling (GC-MS) finds that, as compared with wheat and barley, oat, together with rye, contains relatively higher levels of the most phenolic acids [94].

#### 4.1.6. Fruits and Vegetables

During ripening, fruits undergo significant reprogrammed metabolic changes, profoundly affecting both nutritional and organoleptic qualities. This phenomenon is revealed by metabolic profiling, together with volatile and mineral profiling, using six different platforms (including NMR, GC-MS, and LC-MS), in different melon fruit tissues at three different stages during ripening. This study identifies potential hub metabolites (such as β-carotene, phytoene, and aspartic acid) and mineral element-metabolite interactions, both are important clues in targeted melon breeding for nutrition and flavor improvement [95]. Combined omics including metabolomics study (LC-MS-MS) in 442 tomato accessions show that breeding globally alters tomato fruit metabolomes including flavor volatiles and anti-nutritional compounds steroidal glycoalkaloids. Functional analysis of identified causative genes for steroidal glycoalkaloids shows great potential to reduce steroidal glycoalkaloids in tomato breeding. In addition, *SlMYB12* is identified as an important hub regulator of tomato fruit ripening and metabolism, contributing tremendously to the metabolome-assisted breeding in tomato for nutritional, flavor, and sensory quality [59]. Metabolic profiling (GC-MS and UPLC-MS) together with mineral profiling in raw and cooked potato tubers of 60 unique potato genotypes expands our understandings of the natural metabolic variations of potato tubers, particularly on human health-relevant metabolites, such as carotenoids, chlorogenic acid, vitamins, medicinal substances, and minerals. It provides potato breeding with information for improved health traits of uncooked and cooked foods [73]. Using metabolite profiling (GC-MS) combined with sensory analysis and texture measurement, flavor biomarkers for both desirable attributes such as potato-like and mealy texture (including ethylfuran, 2-pentylfuran, isomenthone) as well as undesirable attributes such as bitter (3,4,5-trimethyl-2-cyclopenten-1-one), earthy (pentan-1-ol), and other off-flavors (5-methylhexan-2-one), were identified in 15 potato genotypes. Those biomarkers pave a way for more effective targeted flavor improvement upon validation [72]. Metabolite profiling (GC-MS and LC-MS) of a potato diversity panel (including both tetraploid and diploid landraces, diploid bred clones, and wild genotypes) indicates clear metabolomic differences between germplasm and highlights which nutritive metabolites are present in potatoes and how they change after cooking. For example, glycoalkaloids and carotenoids decrease while phenolics do not change significantly after cooking [70].

### 4.2. Plant Metabolomics and Quality Evaluation and Authentication of Plant-Derived Beverages

In addition to dietary foods, humans consume many other plant-derived products such as wine, coffee, and tea. Today, metabolomics has been used to evaluate the quality of those typical plant-derived products, to correlate metabolites with taste and health properties in humans, and to promote the production of high-quality health-benefit plant-derived drinks [96,97,98,99,100]. Increasing studies begin to focus on metabolomics responses of humans to these plant-derived beverages as well.

#### 4.2.1. Tea

Metabolomics has been successfully used to evaluate tea quality under normal or stressful conditions and during various processing processes and has constructed metabolic pathways [101]. Metabolite variation in tea is controlled mainly by genetic mechanisms and regulated by environments and agricultural practices, thus profoundly affecting tea quality and flavor. Therefore, there is clear metabolite variation among various tea with different categories and grades [102,103], at different developmental stages [104], and during different manufacturing processes [99]. As a result, metabolic markers identified by metabolomics are important elements for effective evaluation of tea quality under any abovementioned condition. Nevertheless, the genetic basis remains unsolved [105]. A recent study aiming to understand molecular bases for natural variation in catechin compounds in different tea plants reveals that there are not any direct correlations between gene expression levels and metabolite levels, implying likely the existence of posttranscriptional regulatory machineries in tea [106]; however, more studies are needed before such a conclusion can be drawn.

#### 4.2.2. Coffee

Metabolomics has been mainly used for quality evaluation along the value chain in coffee [100,107,108], but with a higher focus on authenticity due to its high economic values [109]. Notably, because there are long-standing concerns about the association of coffee consumption with type-2 diabetes in humans [110], more metabolomics studies have been redirected to understanding the responses of humans to coffee consumption [111,112]. It is clear that coffee intake is associated with widespread metabolic changes and that coffee associated metabolites (particularly lipids) may be used as markers to improve the prediction of diabetes and to understand the health benefits of coffee and tea as well [110,113]. This could be a new direction for future metabolomics studies.

#### 4.2.3. Wine

Various metabolomics technologies have been applied to evaluate and authenticate wine quality worldwide [96,114,115,116,117]. In New Zealand, a juice index database, regarding juice and wine generated from *Sauvignon blanc* grapes grown in different regions over three harvest seasons and fermented with *Saccharomyces cerevisiae* EC1118, is released and available for different users [97]. Similarly, metabolomics has been used to understand the response of humans to wine consumption. Plasma metabolomics analysis with 1157 participants reveals 13 red wine consumption associated metabolites (mainly lipids and organic acids); however, the contribution of them to health or disease has not been assessed [118]. In addition, urine metabolomics analysis shows that red wine intake has a strong impact on overall urinary metabolome, particularly on phenolic microbial metabolites, suggesting its impact on microbiota activity [119]. Interdisciplinary studies are required to fully address the effect of wine consumption on health or disease.

### 4.3. Plant Metabolomics and the Discovery of Natural Plant Products and the Modernization of TCM

Plant-derived natural products are important sources for drugs including TCM development. Metabolomics has emerged as an indispensable tool for drug development to protect human health, which renders drug discovery in plants more efficient and more effective via studying the relationship between medicinal plants and their biological effects [22,120,121,122]. Metabolomics has been used for quality control of natural products by monitoring the efficacy of phytomedicines, such as chamomile [123] and *Cannabis sativa* [124], to ensure efficiency and safety, and content uniformity [21]. Metabolomics has increasingly been applied to detect potential adulterated and contaminated herbal preparations or medicines for key bioactive compounds such as artemisia [125], ginsenosides [126,127], rosavin [128], and others [129], and to detect medicine authentication with multiple botanical origins, such as *Uncariae Rammulus cum Uncis* [130]. Metabolomics has also been used for the standardization of traditional Chinese medicine formulations in *Adhatoda vasica* [131], Xin-Ke-Shu [132], and Huan-Lian-Jie-Du decoction [133]. Meanwhile, metabolomics plays important an role in the understanding of responses of humans to different TCM formulas, such as Xiaoyaosan [134] and Ge-Gen-Qin-Lian decoction [135]. Similar to its applications in wine, tea, and coffee industries, metabolomics would facilitate the standardization of TCM in many aspects, thus, ultimately expanding the international market of TCMs and promoting a healthier global society.

### 4.4. Plant Metabolomics and GM Food Safety

Plant metabolomics has been adapted for the safety assessment of GM crops and derived foods [24,26,136,137,138], albeit that its usefulness is still a hot debate [25,139]. For this purpose, comparative metabolomic analysis between a GM and its non-GM counterpart is performed, and the resulting results usually form the basis for substantial equivalence assessment. Due to the fact that such a one-by-one metabolic comparison is easy to be affected by many factors, metabolomic natural variations are introduced into the assessment system, which provides a feasible safety assessment in GM crops and foods; by comparing the observed metabolic differences between GM and non-GM counterparts with the detected metabolic natural variation of the tested crop species, conclusions regarding GM food safety are reliable, accurate, and convincing [138,140]. It is worth mentioning that metabolomics has been recently recommended for the identification and characterization of genome-edited crops [141]. Different from GM technology, genome editing modifies targeted genes in the genome in a more accurate and precise manner, which cannot be easily detected using methods at the DNA level as it did for GM crops. Therefore, plant metabolomics provides a valuable tool to detect the effects of the newly edited alleles, particularly under the scenery when the regulatory landscape shifts from the process-to product-based characterization [141].

### 4.5. Plant Metabolomics and Plant-Derived Food Safety

Plant protection products protect plants from various micro-/organisms by interfering in metabolic pathways of targeted micro-/organisms. A recent article provides an excellent review on the applications of metabolomics in R&D of pesticides, insecticides, herbicides, and plant growth regulators, and claims that applying metabolomics using proper biological systems helps to elucidate the mode of action of plant protection products and to assess their toxicities, thus, not only accelerating the R&D of green alternative chemicals for plant protection but also assisting regulatory agencies in policymaking regarding their toxicities [142]. Plant metabolomics has recently emerged as a useful tool to study the phenotypic and biological alterations in plant system exposure to various environmental xenobiotics. A recent review summarizes the application of metabolomics in the toxicity assessment of xenobiotics (such as nanomaterials, retardants, pharmaceuticals, and personal care products) and altered levels of environmental nutrients in plants, which highlights the important roles of metabolomics in plant ecological chemistry [143]. Plant metabolomics plays vital roles in the elucidation of heavy metal tolerance in plants and in the identification of metabolic biomarkers for novel heavy metal tolerant crop breeding; those heavy metals include aluminum [144], copper [145], lead and cadmium [146], and zinc [147]. Plant metabolomics finds its use in assessing the health risk of heavy metal accumulation in crops grown under organic and conventional cropping systems, such as in wheat [69].

## 5. Challenges and Future Perspective

**Figure 2:**
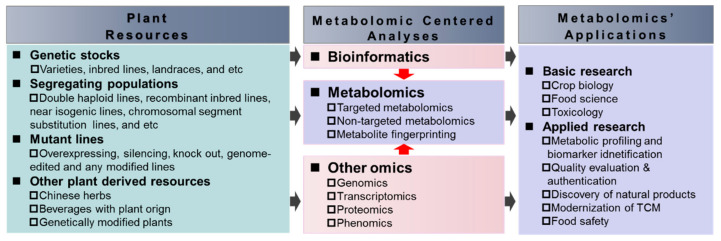
A schematic diagram showing the path of the applications of plant metabolomics in human nutrition and health promotion. TCM, traditional Chinese medicine.

Feeding constantly growing populations under changing climate and deteriorating resource (such as soils and waters) conditions depends extremely on the increase of the production and the decrease of the loss of food products. Meanwhile, preventing overnutrition and malnutrition associated diseases relies largely on the consumption of nutritive and healthy diets. In both cases, plant metabolomics will play essential roles. By elucidating biochemical and genetic bases of traits associated with yield, nutrition, quality, resilience of crops and other plant-derived products including natural products and TCMs, metabolomics will certainly lay a solid foundation for breeding enough nutritive and healthy beneficial foods and plant products to secure food supply, to ensure food quality, and to protect humans from hunger and malnutrition, and from pathogenic diseases (Figure 2).

The link between metabolome (metabolites) and phenome (traits and/or diseases) has been established, however, most of the in vivo functions of metabolites in human nutrition and health remain unknown [76,148,149,150]. This is the most pronounced challenge when plant metabolomics is used to promote human nutrition and health. Increasing metabolomic researches have to be redirected to investigate into the metabolomic responses of human to specific foods or dietary patterns, and to discover biochemical markers associating foods or dietary patterns with diseases, to evaluate effects of known plant-derived nutrients, bioactive compounds (such as polyphenols and bran oils) on human health, and to explore novel health-promoting products. In addition, current plant metabolomics approaches, even combined together, can reveal only a small part of the whole metabolomes in any crops or human cells or tissues [120,148], which restricts the identification of key bioactive compounds associated with human nutrition and health [20,23,54]. Therefore, validation and standardization of individual plant metabolomics pipeline should be carried out to allow the inter- and intra-laboratory exchanges, the establishment of uniform and accessible databases through joint efforts across plant science, food sciences, and human sciences. Moreover, although metabolomics associated with multiple omic studies, such as mGWAS, pGWAS, eGWAS, and mQTL, are effective and powerful, its cost is often well over budget, which hinders the full elucidation of biochemical and genetic regulatory mechanisms underlying crop nutrition traits and genetic diseases. Collaborative studies among different stockholders should be explored to further expand the capacities of metabolomics centered systems biology to promote human nutrition and health.

## 6. Conclusions

Plant metabolomics has significantly advanced our understandings of most of the low molecular weight metabolites that are associated with human nutrition and health in crops and medicinal plants. Plant metabolomics has increasingly been used to establish the association of levels and interactions of several metabolites with specific pathways and traits particularly associated with nutrition or health in important crops and Chinese medicinal herbs. It is time to accelerate crop and plant breeding based on the integration of plant metabolomics with other omics approaches, to promote human nutrition and health by providing improved foods and medicines derived from plants.

## Data Availability

Data is contained within the article.
